# Investigating Changes in the Psychological Health Before and During the COVID Pandemic: A Comparison Study among Turkish Immigrants living in Germany

**DOI:** 10.1192/j.eurpsy.2023.499

**Published:** 2023-07-19

**Authors:** D. E. D. Cindik-Herbrüggen

**Affiliations:** Psychiatry, Neuro-Psychiatrisches Zentrum Riem, Munich, Germany

## Abstract

**Introduction:**

It was stated in other studies that the prevalence of anxiety, depression, and anger increased among the general German population throughout the pandemic (Beutel et al., 2021; Rossi et al., 2020; Smith et al., 2021). Besides, there has been an increase in mental problems among individuals with psychiatric disorders and the immigrant population in society. Migrants are considered a vulnerable group during the outbreak due to low socio-economic status, job losses, and language difficulties.

**Objectives:**

This paper aimed to investigate changes in the psychological health of Turkish immigrants living in Germany during the COVID-19 pandemic. Furthermore, sociodemographic differences as a key factor were analysed in this study. Individuals with lower incomes were expected to suffer more from mental health problems.

**Methods:**

The participants of this research were mainly first and second-generation Turkish immigrants. They were pre-screened for a previous history of mental disorders and screening was performed with SCL-90-R. Of all 177 participants who completed the questionnaire between October 7, 2019, and February 2020, they were recruited again between August 10, 2020, and December 10, 2020, during the pandemic.

**Results:**

According to the findings, a significant difference was found for depression (t=-5.36, p<.001), anxiety (t=-3.01, p<.001), and hostility (t=-3.70, p<.001) between the mean scores of the participants before and during COVID-19 pandemic. It was found that the increase in depression and anxiety symptoms during the coronavirus pandemic was higher among participants with low-income levels (p<.001).

**Conclusions:**

The mental health of our study participants worsened during the current COVID-19 pandemic. Turkish immigrants reported having higher depression, anxiety, and hostility scores in comparison with previous test scores conducted before the outbreak. Participants with low income were at the highest risk for COVID-19-related depression and anxiety.

**Disclosure of Interest:**

None Declared

